# Is mortality truly higher for oncology patients admitted to intensive care units? A matched cohort observational study

**DOI:** 10.1007/s12094-026-04224-9

**Published:** 2026-02-02

**Authors:** Marta Zafra Poves, Maria Angeles Vicente Conesa, Maria Esperanza Guirao García, Manuel Sanchez Cánovas, Nuria Alonso Fernandez, Francisco Ayala de la Peña, Andrés Carrillo Alcaraz

**Affiliations:** 1 Department of Medical Oncology, Hospital General Universitario Morales Meseguer, Av. Marqués de los Vélez, s/n. 30008, Murcia, Spain; 2 University of Murcia, Murcia, Spain; 3 Instituto Murciano de Investigación Biosanitaria, Murcia, Spain; 4 Department of Intensive Care, Hospital General Universitario Morales Murcia, Murcia, Spain; 5 Present Address: Department of Medical Oncology, Hospital General Universitario Gregorio Marañón, Madrid, Spain

**Keywords:** Intensive care unit (ICU), Cancer, Prognosis, Acute mortality, Solid tumors

## Abstract

**Background:**

Cancer patients often develop life-threatening events that prompt intensive care unit (ICU) admission. However, uncertainty regarding prognosis may hinder timely referral. We compared ICU survival in adults with solid tumors admitted emergently for medical or urgent surgical reasons with that of non-cancer controls.

**Methods:**

We retrospectively analyzed 167 consecutive adults with solid tumors emergently admitted to a mixed ICU in a single center between 2010 and 2016, and compared them with two propensity-matched non-cancer cohorts. We made two 1:1 comparisons: (1) cancer and non-cancer patients matched for age, sex and do-not-intubate order; (2) the same cancer cohort matched additionally for admission diagnosis, maximum SOFA, SAPS II and Charlson Comorbidity Index. Primary outcome was ICU mortality; hospital mortality and 90-day survival were secondary endpoints.

**Results:**

Cancer cases represented 4.8% of all ICU admissions; 54% had metastatic disease, 41% acute respiratory failure, and 28.7% sepsis/shock. When matched only for demographic and functional factors, cancer patients had higher intensive care unit and hospital mortality rates than controls (27.5% vs 10.8%, *p* < 0.001, and 35.3% vs 16.2%, *p* < 0.001, respectively). After matching for severity and comorbidity, ICU and hospital mortality no longer differed significantly (27.5% vs 19.8%; *p* = 0.094, and 35.3% vs 28.7%; *p* = 0.4). 90-day survival was significantly lower for cancer patients (64.7% vs 80.2%, *p* < 0.001), but no differences were found with controls matched for severity and comorbidity (64.7% vs 71.3%, *p* = 0.4).

**Conclusions:**

Solid-tumor patients admitted to the ICU are generally more severely ill and thus present higher crude mortality than non-cancer patients. However, when severity and comorbidity are equivalent, outcomes are similar. Therefore, intensive care should be offered to cancer patients with reversible critical illness and acceptable baseline status, and a cancer diagnosis alone should not be considered a contraindication for ICU admission.

## Background

The high incidence of cancer in our setting, along with decreasing mortality rates for many malignancies, has led to a progressive increase in the prevalence of cancer [1]. As a result, patients live more time with cancer and are exposed for extended periods to oncologic treatments. Improved survival is associated with a higher likelihood of experiencing a severe acute event requiring intensive care unit (ICU) admission, whether due to cancer-related complications, treatment-related toxicities, or unrelated conditions linked to age-associated comorbidities. Approximately 15% of ICU beds in Europe are occupied by patients with cancer, with progressively better short-term survival and mortality rates around 27% [2, 3]. Oncology patients have also benefited from advances in critical care, such as early and optimized management of sepsis, use of non-invasive mechanical ventilation (NIMV), and the application of organ support therapies [4].

However, when a patient with cancer develops a severe acute condition, cancer diagnosis frequently acts as a determinant in ICU admission decisions, sometimes irrespective of acute illness severity. While recognizing the complexity of decision-making in emergency settings, the mere presence of advanced cancer should not, by itself, preclude ICU admission, as supported by several studies [5–7].

Numerous reports indicate that ICU short-term outcomes among cancer patients varies depending on the time of study [3, 8, 9], ICU type and admission policies [10], admission cause (medical vs. surgical), cancer type (resectable or metastatic, solid tumors vs. hematologic malignancies, stem cell transplant recipients), and the type of organ support applied, with survival rates ranging widely from 12 to 48% [8, 9, 11–17]. Survival outcomes are generally lower for medical admissions compared to elective surgical cases, as the former are typically more critically ill and include patients admitted for cancer-related complications, who have the worst prognosis [10–13, 15–18]. The intensity of support also influences outcomes and must be considered alongside ICU-specific policies regarding the implementation or withdrawal of intensive treatment [19, 20].

Another key factor impacting mortality is the distinction between patients with solid tumors and those with hematologic malignancies. Hematologic malignancies are typically associated with greater baseline immunosuppression and may involve stem cell transplantation, contributing to worse ICU outcomes. Despite this, most studies evaluating cancer patients in the ICU include both groups, further increasing the heterogeneity of findings and limiting comparability across studies [10, 14].

There are few studies directly comparing ICU patients with and without cancer. Most large cohort studies include both populations, often with a predominance of surgical cases. For instance, the European multicenter SOAP study [13], which included 3147 ICU patients with sepsis (15% of whom had cancer, 85% with solid tumors), found comparable illness severity, higher surgical admission rates, and similar in-hospital mortality between patients with solid tumors and those without cancer (27%). A prospective U.S. cohort study (2002–2011) analyzing 4330 ICU patients with solid tumors found similar clinical characteristics and illness severity scores to patients without cancer, in contrast to hematologic patients [8]. A retrospective European study of ICU patients aged ≥ 65 years (2009–2014) reported similar mortality rates between 332 patients with solid tumors and 262 without (33.6% vs. 32.7%) although cancer patients were younger and had higher SAPS II scores [21].

In Spain, the ELVIN-HELICS registry-based study compared ICU outcomes for patients with and without cancer [11]. Oncology patients were older, more often admitted from inpatient wards, and more likely to have surgical reasons for admission. They presented with higher severity scores on admission, shorter ICU stays, and higher ICU mortality (12.3% vs. 8.5%; *p* < 0.001). However, the heterogeneity of these cohorts—combining medical and elective surgical cases, hematologic and solid tumors—limits the evidence regarding true mortality among solid tumor patients experiencing an acute critical illness that prompts ICU consideration.

Therefore, as the main outcome of our project, we have performed a study focusing solely on patients with solid tumors, excluding those admitted exclusively for postoperative monitoring, and comparing outcomes with critically ill patients without cancer. This may offer clearer insights into the prognosis of cancer patients in the ICU. Such data guide evidence-based decisions on ICU admission for cancer patients with acute critical illness.

## Methods

### Study design and patient selection

We performed a single-center, retrospective observational study in the medical–surgical ICU of a Spanish tertiary hospital. Consecutive adults (≥ 18 years) with histologically confirmed—or ICU-diagnosed—solid tumors admitted emergently for medical or unplanned surgical reasons between 1 January 2010 and 31 December 2016 were included. Admission decisions were made jointly by the ICU and Oncology teams, each staffed by an in-house physician 24/7 throughout the study period.

Exclusion criteria were: hematologic malignancy, elective postoperative monitoring, complications of scheduled surgery, ICU readmission during the same hospitalization, transfers from other hospitals without continuity of care at our center, and localized solid tumors treated definitively with surgery or radiotherapy when the ICU admission was unrelated to the cancer or its treatment.

### Control cohorts

Control patients without cancer were selected from all medical admissions to the same ICU during the study period. Two propensity-matched cohorts (1:1) were created:

- Clinical profile match—matched exactly for age, sex, and do-not-intubate (DNI) order status.
- Severity score match—matched exactly for age, sex, DNI order status, admission diagnosis, maximum Sequential Organ Failure Assessment (SOFA), Simplified Acute Physiology Score II (SAPS II), and Charlson Comorbidity Index (CCI).

### Data collection

General clinical, functional, prognostic, and comorbidity variables were recorded prospectively for all ICU admissions as part of routine practice (demographic and anthropometric data, clinical data from admission to ICU, type of support in the ICU, and ICU score data). Oncologic characteristics and follow-up data were collected retrospectively by a medical oncologist, who exhaustively reviewed the electronic medical record (data related to cancer treatment, and oncological disease).

Treatment intent is a nominal qualitative variable defined as the actual intent based on objective criteria with which the responsible oncologist proposes the cancer treatment that the patient receives or has received. It can be curative (with a high probability of cure), control (whose objective is to achieve a response to the disease, with potential benefit in survival, although not cure) or palliative (the objective of treatment is to control symptoms derived from the disease, without a probability of increased survival demonstrated in clinical trials).

Tumor status is an ordered qualitative variable defined as the state of the neoplastic disease at a given time, in this study upon admission to the ICU. Based on the estimate of the responsible oncologist from the disease data. According to this classification, a distinction is made between patients in whom the cancer has been removed and there is no recurrence in the imaging studies performed, also described as “no evidence of disease” (NED), those who have a potentially curable disease (through local treatments involving surgery and/or radiotherapy in addition to systemic treatment in some cases), and those who have a neoplastic disease with no curative treatment options, but with the intention of increasing survival or controlling symptoms.

### Statistical analysis

Categorical variables are presented as counts and percentages; continuous variables as mean ± SD or median (IQR), depending on normality. Group comparisons used the *χ*^2^ test for categorical variables, Student’s t test for normally distributed continuous variables, and the Wilcoxon signed-rank test for non-parametric data. Two-tailed *p* values ≤ 0.05 were considered statistically significant.

The primary endpoint was ICU mortality. Secondary endpoints were in-hospital mortality and 3-month overall survival. To evaluate the impact of a cancer diagnosis on ICU mortality, we first performed exact-matching propensity analysis controlling for age, sex, and DNI order status. A second analysis compared the cancer cohort with the severity-matched control cohort described above.

Propensity score matching was performed using a nearest-neighbor model without replacement, with a 1:1 ratio. To determine the effectiveness of propensity score matching for controlling the differences between groups, standardized mean differences (SMDs) were calculated for each variable before and after matching. SMDs less than 10% indicated successful propensity scores matching and balancing between the two groups. For comparisons in the matched cohorts, Student’s paired t test, Wilcoxon signed-rank test, and McNemar test were used. Univariate comparisons of survival between groups were performed using log-rank tests and univariate Cox models for estimating HR and its 95% confidence interval.

Based on an expected 20% mortality in controls, a sample of 296 patients (148 per group) was required to provide 80% power to detect a 10% difference in ICU mortality at *α* = 0.05.

Analysis was conducted with IBM SPSS Statistics, version 25. This study was approved by the institutional Research Ethics Committee of Morales Meseguer University General Hospital (EST: 40/19).

## Results

### Cohort characteristics

**Fig. 1:**
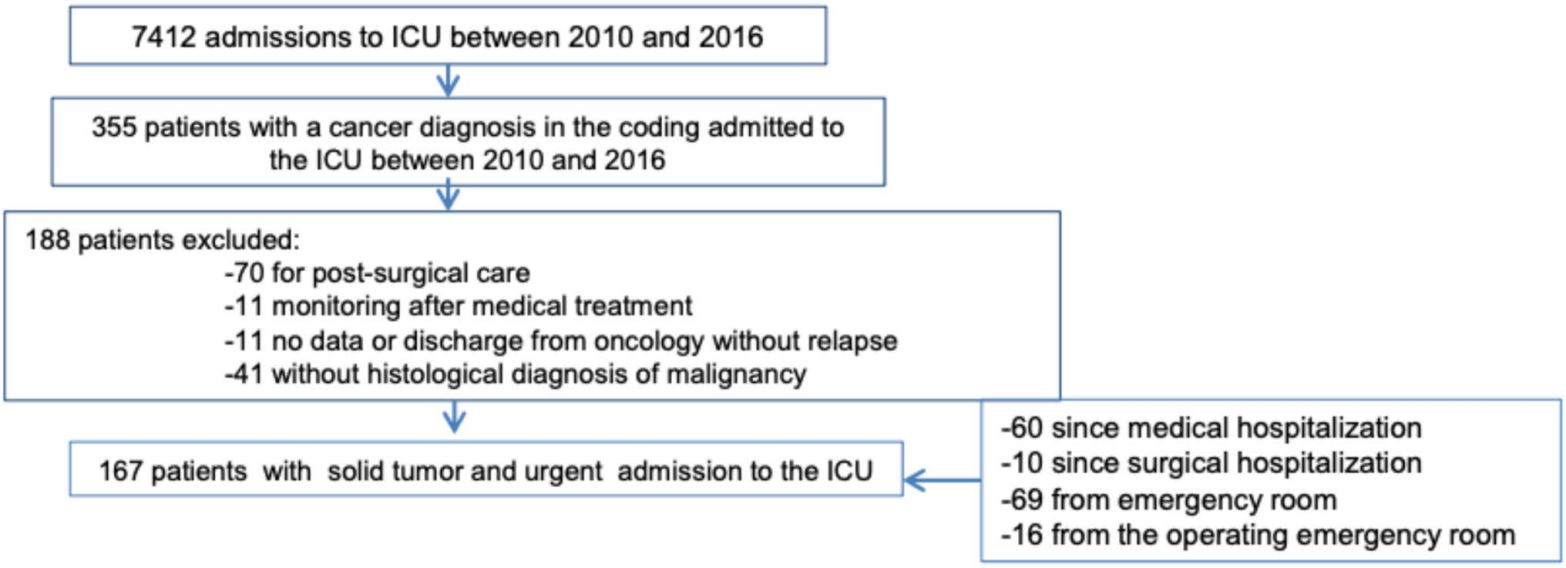
Cohort selection. Consort diagram for the cancer cohort (*n* = 167)

Between 2010 and 2016, there were 7412 admissions to the ICU of Morales Meseguer Hospital; 355 of them involved patients with solid cancer. Of these, 167 consecutively admitted adults with solid tumors met the study criteria (Fig. 1).

Baseline demographic and clinical data are summarized in Table 1. Most patients were male (67.7%) and functionally independent (ECOG 0–1, 62.3%), with a median age of 64 years. Pulmonary/pleural primary tumor predominated (31.7%). The median Charlson Comorbidity Index (CCI) was 6 (IQR 4–7).

**Table 1:** Baseline demographic and clinical data

Characteristics *N* (%)		*N* (%)
Age (years)	Mean (SD)	62 (13)
	Median (range)	64 (18–83)
Sex	Male	113 (67.7)
	Female	54(32.3)
Baseline functional status	ECOG 0–1	104 (62.3)
	ECOG 2	48 (28.7)
	ECOG 3–4	15 (9)
Charlson Index	Median (RIQ)*	6 (4–7)
Previous comorbidity	COPD (chronic obstructive pulmonary disease)	24 (14.4)
	Diabetes Mellitus	40 (24)
	Cirrhosis	4 (2.4)
Reason for admission	Acute respiratory failure	67(40.1)
	Sepsis/Shock	48 (28.7)
	Cardiac reason	24 (14.4)
	Abdominopelvic pathology	17 (10.2)
	Acute renal failure	5 (3)
	Others	6 (3.6)
Acute complications during admission	Acute renal failure	64 (38.3)
	Immunosuppression	27 (16.2)
	Neutropenia	29 (17.4)
Type of primary tumor	Pleuropulmonary	53 (31.7)
	Colorectal	24 (14.4)
	Breast	24 (14.4)
	Upper digestive and hepatobiliary	15 (9)
	Head and neck	15 (9)
	Genitourinary	13 (7.8)
	Gynecologic	9 (5.4)
	Other	13 (7.8)
Tumor Stage	I–II	22 (13)
	III	55 (33)
	IV	90 (54)
Overall cancer status	No evidence of disease	32 (19)
	Potentially curable	45 (27)
	Incurable	90 (54)

Cancer treatment intention was potentially curative in 71 patients (42.5%), disease-control in 76 (45.5%), and strictly palliative in 20 patients (12.0%), who were receiving systemic treatment to control their cancer symptoms, or supportive care alone. At ICU admission, 27.5% were receiving first-line therapy for advanced disease, 25.1% were on neoadjuvant or radical treatment, 12% were on second- or later-line therapy, and 18% were treatment-naïve (new diagnosis or relapse).

The most common reason for ICU admission was acute respiratory failure (40.1%), followed by sepsis/shock (28.7%). ICU mortality was 27.5%; a further 16 deaths occurred on the ward, yielding an overall in-hospital mortality of 35.3%. 90-day survival after ICU discharge was 64.7%.

### Comparison with controls matched for age, sex, and do-not-intubate order

The cancer cohort was first matched 1:1 with ICU patients without cancer for age, sex, and DNI order status **(**Table 2**).**

**Table 2:** Characteristics of the three cohorts: patients with cancer, matched patients without cancer and severity-matched patients without cancer (*n* = 167 each)

	Cases	Controls without cancer	*p* value	Controls without cancer matched for severity	*p* value
Age, median (range)	62 (13)	62 (13)	1	62 (13)	1
Sex (Male)	113 (67.7)	113 (67.7)	1	113 (67.7)	1
DNI order status	40 (24%)	40 (24%)	1	40 (24%)	1
SAPS II Mean (SD)	47.17 (16.1)	33.82 (16.8)	< 0.001	47.65 (15.2)	0.780
Charlson Index (CCI) Median (IQR)	6 (4–7)	2 (1–4)	< 0.001	5 (4–7)	0.236
Reason for admission			0.455		1
Acute respiratory failure	67 (40.1)	60 (35.9)		67 (40.1)	
Sepsis/Shock	48 (28.7)	40 (24)		48 (28.7)	
Cardiac	24 (14.4)	26 (15.6)		24 (14.4)	
Abdominal–pelvic pathology	17 (10.17)	20 (12)		17 (10.17)	
Acute renal failure	5 (3)	11 (6.6)		5 (3)	
Others	6 (3.6)	10 (6)		6 (3.6)	
Previous pathology					
COPD	24 (14.4)	25 (15)	0.877	46 (27.5)	0.003
Diabetes	40 (24)	57 (34.1)	0.040	83 (49.7)	< 0.001
Cirrhosis	4 (2.4)	8 (4.8)	0.240	12 (7.2)	0.040
Acute Kidney Disease	64 (38.3)	46 (27.5)	0.036	67 (40.1)	0.737
Independence baseline activities daily living	117 (70.1)	128 (76.7)	0.361	120 (71.9)	0.941
Neutropenia	29 (17.4)	0 (0)	< 0.001	0(0)	< 0.001
Previous Antibiotics	85 (50.9)	29 (17.4)	< 0.001	33 (19.8)	< 0.001
Maximum SOFA Mean (SD)	7.75 (4.6)	4.50 (4.6)	< 0.001	8.15 (3.6)	0.388

**Fig. 2:**
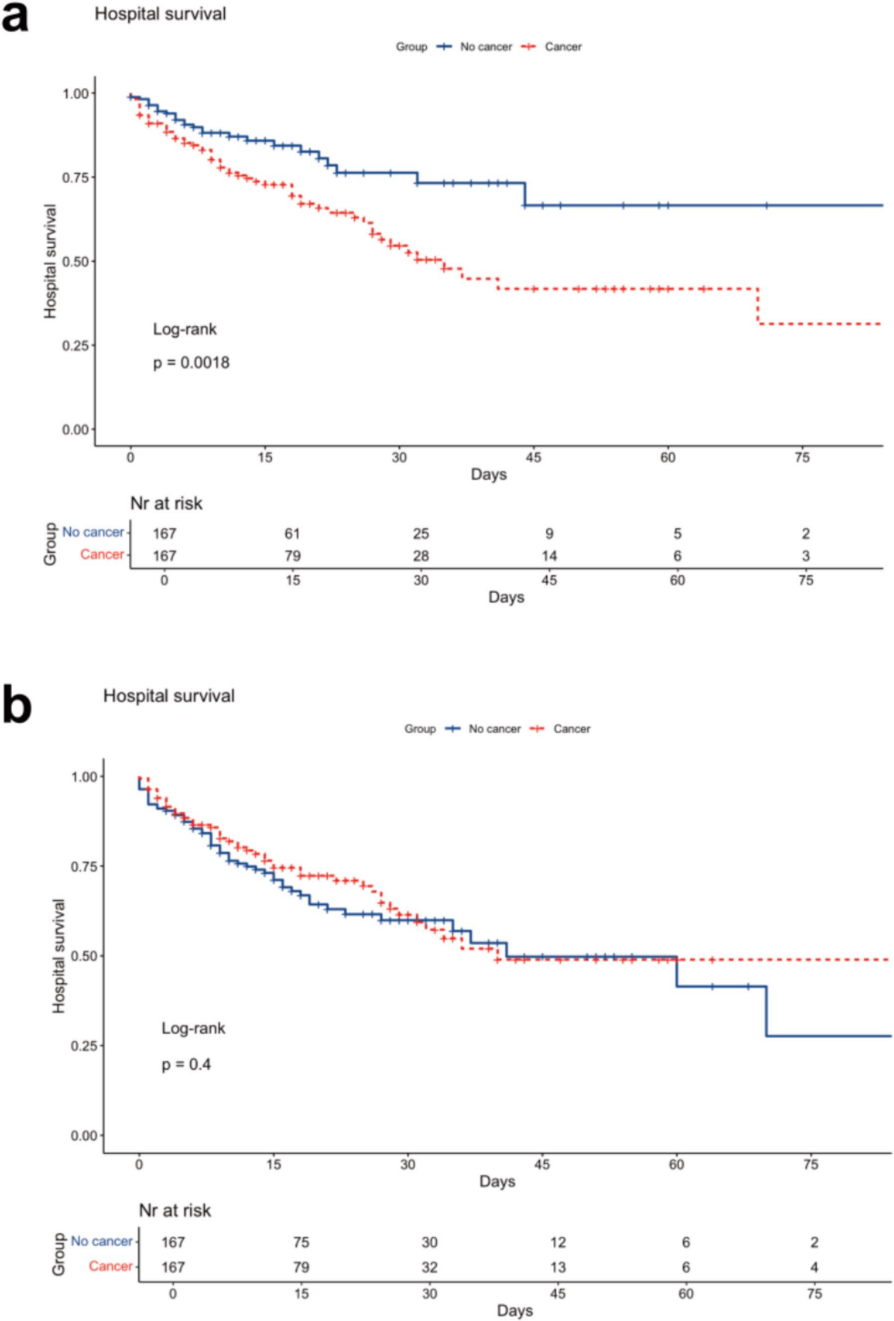
Hospital survival in cancer and control cohorts. Kaplan–Meier survival curves comparing **a** the cancer cohort and the control group matched for age, sex, and DNI status; and **b** the cancer cohort and the control group matched for age, sex, DNI status, and illness severity

Cancer patients had significantly higher ICU mortality (27.5% vs 10.8%; *p* < 0.001) and in-hospital mortality (35.3% vs 16.2%; *p* = 0.0018). (Fig. 2a). ICU length of stay was longer in cancer patients (median 4 days vs 2 days; *p* = 0.023), whereas total hospital stay did not differ (median 13 vs 10 days; *p* = 0.230). 90-day survival was lower in cancer patients (64.7%) in comparison with controls (80.2%), and this difference was statistically significant (HR 2.7; 95% CI 1.8–3.9; *p* < 0.001).

Severity scores (maximum SOFA, SAPS II) and CCI were higher in the cancer group. Diabetes, acute kidney injury, neutropenia, and prior antibiotic exposure were also more frequent. Reasons for admission were similar between groups. **(**Table 2**).**

**Fig. 3:**
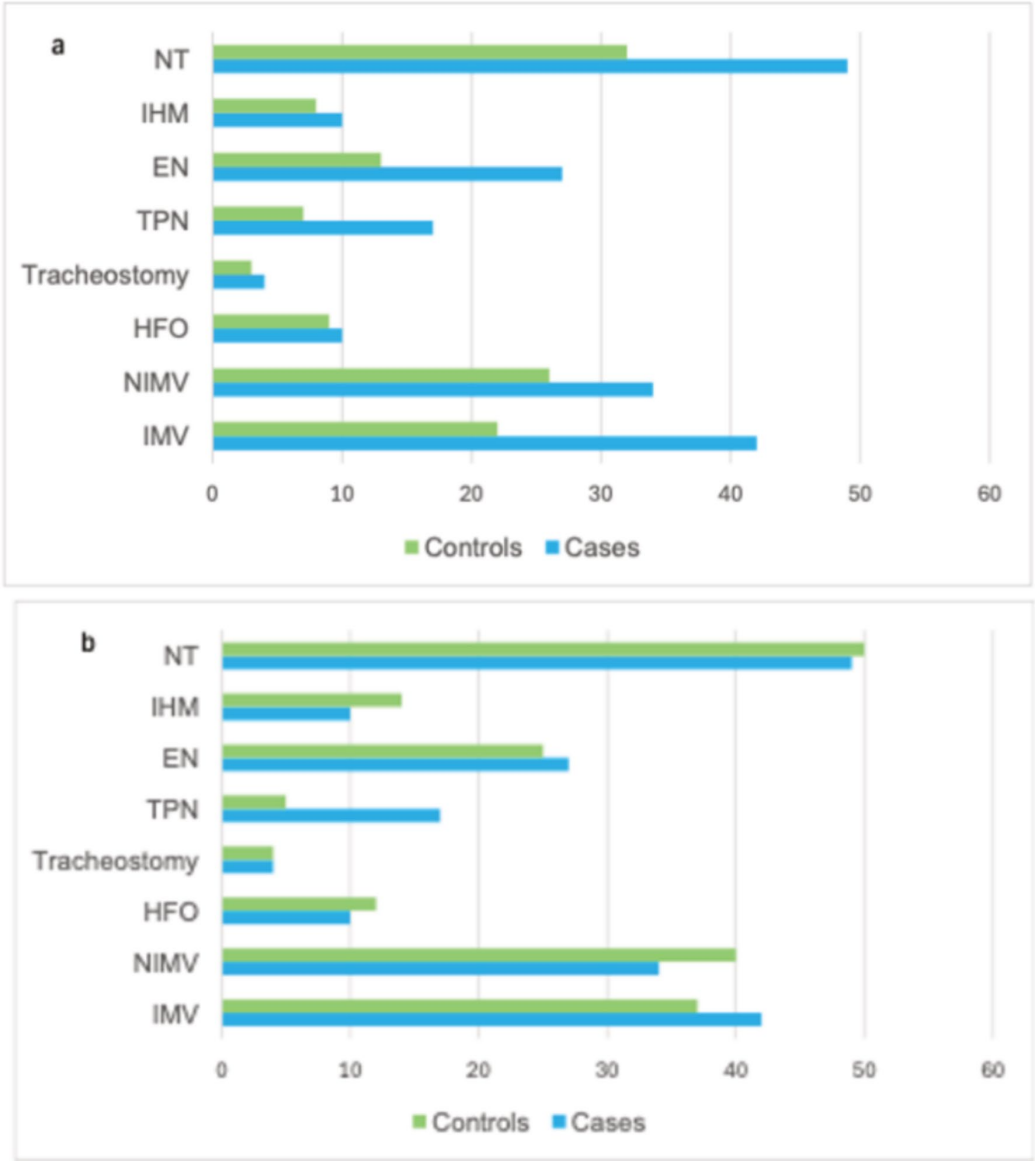
Comparison of organ support measures between cohorts of ICU patients. **a** Comparison of patients with and without cancer. **b** Comparison of cancer patients and severity-matched controls. EN: enteral nutrition; HFO: high-flow oxygen; IHM: invasive hemodynamic monitoring; IMV: invasive mechanical ventilation; NIMV: non-invasive mechanical ventilation; NT: nasogastric tube; TPN: total parenteral nutrition

With respect to organ support, cancer patients required total parenteral nutrition (17.4% vs 6.6%; *p* = 0.002), enteral nutrition (26.9% vs 13.2%; *p* = 0.002), nasogastric tube placement (48.5% vs 32.3%; *p* = 0.003), and invasive mechanical ventilation (41.9% vs 21.6%; *p* < 0.001) more frequently. Use of high-flow oxygen, non-invasive ventilation, and invasive hemodynamic monitoring was comparable **(**Fig. 3a**).**

Regarding the group of palliative patients (*n* = 20) admitted to ICU, the main causes of admission were sepsis (60%), cardiac event (20%), respiratory failure (15%), and kidney failure (5%). Furthermore, in four of these patients, admission to the ICU was due to a complication related to the onset of cancer. Their subsequent deterioration prevented them from receiving oncological treatment. Globally, their acute prognosis was the same than that of the rest of patients, with 90-day survival of 65%.

### Comparison with controls matched for illness severity

To test whether cancer independently influenced short-term outcomes, we created a second 1:1 control cohort matched for age, sex, DNI order status, admission diagnosis, maximum SOFA, SAPS II, and CCI. ICU survival was 72.5% in cancer patients and 80.2% in controls (*p* = 0.094). In-hospital mortality was 35.3% vs 28.7% in controls, (*p* = 0.4). **(**Fig. 2b**)**. ICU and hospital lengths of stay were similar. 90-day survival was 64.7% vs 71.3% again without statistical significance (HR 1.26; 95% CI 0.86–1.85; *p* = 0.4).

Controls had higher rates of COPD, diabetes, and cirrhosis, whereas cancer patients more often received prior antibiotics and were neutropenic. Acute kidney injury and baseline independence in activities of daily living did not differ between cancer patients and controls **(**Table 2**).**

During the ICU stay, cancer patients required total parenteral nutrition more often (17.4% vs 5.4%; *p* = 0.001) and a longer duration of invasive hemodynamic monitoring (mean 4 vs 2 days; *p* = 0.005). Requirements for mechanical ventilation (invasive, non-invasive, or high-flow oxygen nasal cannula), enteral nutrition, and nasogastric tube were similar (Fig. 3b**).** Cancer patients received more red blood cell (26.3 vs 3%, *p* < 0.001) and platelet transfusions (9 vs 0.6%, *p* < 0.001), with no difference in nosocomial infection rates (7.2% vs 11.4%; *p* = 0.187).

## Discussion

Here we analyzed all emergency ICU admissions of adults with solid tumors over seven years in a single center, and compared outcomes with two non-cancer cohorts: first, matched for age, sex, and DNI order status; second, additionally matched for admission diagnosis and severity scores. In the age/sex/DNI order status-matched analysis, cancer patients showed higher mortality, driven by higher SOFA and SAPS II scores. When severity and comorbidity were also balanced, a cancer diagnosis per se no longer predicted excess mortality.

Regarding the study setting and cohort selection, our hospital employs 24/7 in-house oncologists who pre-screen candidates for ICU transfer, ensuring good pre-morbid function and a realistic expectancy of benefit. Such triage filters, plus close communication with intensivists, likely reduce futile admissions. Unlike reports from dedicated cancer centers [16, 22–26], with different criteria for ICU admission, our mixed medical–surgical ICU reflects typical tertiary general hospital practice [14, 27], enhancing external validity and clinical applicability of our results.

We deliberately excluded hematologic malignancies and elective postoperative monitoring. Previous studies often mix solid and hematologic tumors [5, 7, 9, 13, 14, 20, 22, 23, 25], focus on single cancer types, such as lung cancer [28, 29], include postoperative surveillance cases with better prognoses [2, 3, 8, 10–12, 22], or are literature reviews rather than original cohorts [9, 12, 30]. Our homogeneous cohort, unplanned admissions for solid tumors, mostly medical (> 90%), and predominantly metastatic disease (54%), captures the current oncology case mix in general ICUs and counters the outdated notion that intensive care should be reserved for patients with resected or curative-intent cancers alone [5–7]. Acute respiratory failure (41%) and sepsis/shock (28.7%) were the leading reasons for admission, mirroring national registry data [11]. ICU mortality (27.5%) and overall hospital mortality (35.3%) align with recent Western series reporting 27–44% [8, 12, 13, 21, 26]; Spanish figures range 27–36% [11, 14]. Thus, our work reflects current practice and daily decision-making about critically ill oncology patients.

In the age/sex/DNI order-matched comparison, cancer patients had greater physiologic derangement, higher comorbidity, and three times higher ICU mortality (27.5% vs 10.8%). These findings confirm that cancer often coexists with more severe acute illness, a conclusion also supported by the absence of differences in cause of ICU admission between cases and controls. Matching for severity, together with cause of admission and comorbidity, however, abolished the mortality gap and equalized ICU and hospital length of stay. Thus, although cancer is generally associated with greater clinical severity at the time of ICU admission, when patients are matched for severity of illness and comorbidity, the presence of cancer does not translate into higher ICU mortality, provided that appropriate and individualized critical care support is offered. In our study, both groups had the same proportion of patients with DNI order (24%), suggesting comparable limitations of care. Therefore, a cancer diagnosis alone should not be used as a justification for denying ICU admission or for reducing the intensity of life-sustaining treatments in patients whose baseline functional status makes them eligible for and potentially responsive to intensive interventions.

Only a few studies closely resemble our design. In the Spanish ENVIN-HELICS registry [11], cancer patients were older and predominantly admitted for surgical reasons, making them clinically dissimilar to ours, yet they still showed higher severity scores and mortality. The SOAP study reported comparable severity and mortality among septic ICU patients with and without cancer, although a higher mortality was reported in oncologic patients with three or more organ failures [13]. A U.S. cohort analysis [8] found that solid-tumor patients had severity scores comparable to non-cancer patients but higher mortality, alongside less frequent use of vasopressors and mechanical ventilation. In our first matched comparison, invasive hemodynamic monitoring was similarly uncommon (< 10%), but cancer patients required invasive ventilation nearly twice as often (around 40%), with no difference in non-invasive ventilation. In the severity-matched analysis, use rates for both hemodynamic monitoring and ventilation (invasive or non-invasive) converged, and acute mortality remained equivalent. These data also support individualized admission decisions rather than disease-based exclusion.

The main limitation of the study was its single-center and retrospective nature: one possible solution would be to conduct a multicenter, prospective study, especially if it included hospitals of different levels of complexity to analyze whether there are differences in decision-making and the prognostic evolution of the studied population. Additional limitations should be acknowledged: importantly, there are no data on oncology patients who were evaluated but ultimately denied ICU admission, which may introduce a major selection bias in the interpretation of real mortality, a topic for futures prospective studies. The cohort combines heterogeneous solid tumor types (lung, colorectal, breast, gynecologic, etc.) with very different prognoses and complications, which could dilute or mask tumor-specific differences. Moreover, the study reflects admissions from 2010–2016, and results may not fully represent current practice, in the current state of immunotherapy and targeted therapies. Functional and quality of life outcomes after discharge from the ICU were not reported in this report, although they were collected. Finally, the model of care with 24/7 in-house oncologists is not universal, which restricts extrapolation to other settings.

Key strengths of our study include a sufficiently sized, clinically relevant, and homogeneous cohort of patients with solid tumors admitted emergently, predominantly for medical reasons, and not for elective postoperative care. This population reflects the type of patients who often present the greatest uncertainty during on-call decision-making in intensive care and oncology. Coupled with our dual propensity score analysis, which exclude mortality differences greater than 10% attributable solely to cancer, these features enhance both the internal validity and the practical relevance of our findings.

In conclusion, hospital mortality following ICU admission is higher in cancer patients compared to non-cancer patients when matched for age, sex, and do-not-intubate order status. However, when both groups are also matched for illness severity and comorbidity, ICU and hospital mortality no longer differ significantly. These findings indicate that although cancer patients admitted to the ICU are generally more severely ill, when severity is comparable, outcomes are similar to those of non-cancer patients. The clinical profile of solid tumor patients requiring urgent ICU admission, outlined in this study, poses a significant challenge for both intensivists and oncologists, who must assess the potential for recovery despite high severity. Our results support that, once ICU admission is jointly agreed upon, critically ill cancer patients can benefit from intensive care to the same extent as those without cancer. Therefore, when appropriately selected through multidisciplinary evaluation, intensive support should not be viewed as excessive treatment, but rather as a necessary component of high-quality oncologic care.

## Data Availability

The data that support the findings of this study are available from the corresponding author, upon reasonable request and institutional permission.
